# Challenges faced by community connectors: lessons learned from a Taiwan public health initiative

**DOI:** 10.1186/s12877-022-03565-8

**Published:** 2022-11-19

**Authors:** Lee-Fen Ni, Shu-Ying Lo, Shu-Li Chia, Chao-Chun Wu, Fen-Fang Chung, Yu-Hsin Wang, Ping-Ru Hsiao, Chia-Ling Lin, Xaviera Xiao, Chiu-Tzu Lin, Li-Fen Chao

**Affiliations:** 1 Department of Nursing, Chang Gung University of Science and Technology and Linkou Chang Gung Memorial Hospital, Taoyuan City, Taiwan, Republic of China; 2 Health Promotion Administration, Ministry of Health and Welfare, Taipei City, Taiwan, Republic of China; 3 Chang Gung University of Science and Technology, Taoyuan City, Taiwan, Republic of China; 4 Department of Nursing, Chang Gung University of Science and Technology, Taoyuan City, Taiwan, Republic of China; 5 Clinical Competency Center, Chang Gung University of Science and Technology, Taoyuan City, Taiwan, Republic of China; 6 Department of Nursing and Lecturer, Linkou Chang Gung Memorial Hospital and Chang Gung University of Science and Technology, Taoyuan City, Taiwan, Republic of China; 7 Clinical Competency Center, Department of Nursing, Chang Gung University of Science and Technology, Taoyuan City, Taiwan, Republic of China; 8 Department of Emergency Medicine, Linkou Chang Gung Memorial Hospital, Taoyuan City, Taiwan, Republic of China

**Keywords:** Community connectors, Public health workers, Older adult health, Content analysis

## Abstract

**Background:**

Effective solutions that meet the diverse community health needs of older adult populations are of critical importance. To address these needs, a nationwide community connector team—tasked with providing referral support to older adult populations and completing an asset mapping resource inventory initiative centered around the needs of older adult populations—was developed in Taiwan. The purpose of this qualitative study was to explore community connectors’ experiences and challenges.

**Methods:**

Community connectors (*n* = 26) across four diverse sites participated in focus group interviews in July 2020. Interviews explored the challenges community connectors encountered in their roles; the strategies used to address these challenges; the asset mapping process; and on how they conceptualized their roles. Qualitative content analysis was applied.

**Results:**

Three themes were uncovered: developing community ties, cross-organization interactions and professional conflicts. The findings show that community connectors face hurdles in uncovering community resources and that they experience considerable professional instability. The findings also shed light on the day-to-day approaches used to navigate on-the-job challenges and the steps taken to develop community partnerships.

**Conclusions:**

The experiences of community connectors provide important insights and can serve to illuminate the development of similar initiatives that seek to use community connectors for community health related purposes.

**Supplementary Information:**

The online version contains supplementary material available at 10.1186/s12877-022-03565-8.

## Introduction

In discussion of best practices to support community health, the importance of championing existing community-led organizations and local resources has been argued from the perspective of improved health outcomes, stronger community engagement and reduced healthcare reliance [1–3]. However, the potential, resiliency and diversity of community resources are often not fully detectable to community members; in some instances, these resources may not be utilized to their full capacity [4]. Moreover, lack of access to relevant resources can be further compounded for those who are older adults, hold marginalized identities and reside in rural settings [5]. Notably, the challenges involved in providing adequate community-based care to older adult populations has been recognized as a major public and community health concern by the World Health Organization [6, 7]. Indeed, older adult populations have complex and varied needs [8, 9]. Beyond navigating the physical health consequences of aging, issues relating to self-care and domestic activities, social isolation, and mobility related challenges can lead to reduced mental health and wellbeing.

Globally, the task of ensuring that the healthcare and social wellness needs of older adult communities are met has become a pressing issue, given shifting demographic trends [10]. Like many middle- and high-income countries, Taiwan faces challenges that come with supporting a considerable ageing population. Notably, Taiwan is projected to become a super-aged society by 2026 [11]. The formal resources to meet this population’s health and wellness needs are often not commensurate and, in some instances, are simply inadequate [12]. This is particularly true amongst ethnically and geographically marginalized older adult communities [13]. When considering the diversity of challenges and complexities involved in providing appropriate care and support to facilitate healthy aging, it has been argued that a multipronged approach to support older adult populations' health and wellness is needed [14, 15].

In 2019, as part of an initiative to support Taiwan’s aging population, the Taiwan Ministry of Health of Welfare spearheaded the development of a nationwide Resource Integration Hub, referred to as the HUB project [16]. The project consists of 101 community connectors (hereon referred to as connectors) working across urban, rural and semi-urban sites. Indeed, community connector initiatives have been developed in other countries. By and large, these programs typically aim to take advantage of community connectors’ ability to engage with underreached communities, however the specific aims and protocols of these initiatives will vary based on community needs [17, 18]. In the case of the HUB project, connectors receive introductory as well as on-going training. Connectors were familiar with the local healthcare landscape, given their previous work experience in public health fields or as healthcare professionals. In their roles, connectors are tasked with two major directives. Firstly, connectors collect data on local organizations and support systems for the purposes of developing a resource database for older adult populations. Connectors are responsible for compiling information on resources related to ten domains covering aspects of physical health, social inclusion, and wellbeing. These domains include recreation, home safety and fall prevention; geriatric nutrition; dementia friendly communities; chronic disease management; preventative care; transportation; social inclusion; welfare and benefits; and miscellaneous. Secondly, connectors provide resource referrals and support to service users who are unable to seek out adequate and relevant services, such as access to transportation services or nutrition assistance programs.

In order to collect relevant data, connector teams follow an asset mapping process. Asset mapping, underpinned by a strength-based approach to community resilience and development, seeks to uncover and tap into the various and diverse resources that already exist within communities [4]. While a needs assessment approach to community development may analyze data to conceptualize problems, an asset mapping approach seeks to understand what members of the community would identify as a problem that needs to be addressed [19]. By that same measure, solutions are also derived by uncovering the existing strengths and resources within the community. Namely, an asset mapping approach is focused on understanding how existing resources, formal and informal, can be supported, adapted and utilized.

The study sought to answer two foundational questions:

1. What are the challenges that community connectors face in their roles?
2. How do community connectors approach the demands of their roles?

## Methods

### Study design

An exploratory qualitative study design consisting of focus group discussions was used. The focus group discussions provided an opportunity for connectors to discuss how they conceptualized their roles, the asset mapping process and the challenges they faced.

### Participants and setting

The focus group discussions were conducted across four HUB sites. The site selection process involved categorizing potential sites based on geography (e.g., north, west) and setting (e.g., urban, rural), and making a random selection amongst the different categories. This random selection process was used to make sure that the four sites would capture the diverse settings of the HUB initiatives. To ensure anonymity, these four sites have been referred to as Site A, B, C and D. Site A is a small semi-urban city located in western Taiwan. Site B is a rural area located in northeast Taiwan. Site C is located in a highly urban area of northern Taiwan. Finally, site D is a rural area located on the southeast coast of Taiwan. Connectors across the four sites were invited to participate over email. Eligibility criteria included connectors who had participated in the developed community connector training program and who were willing to provide consent to participate in the focus group. In total, there were 26 connectors working across the four sites. Ultimately, all connectors were recruited, with each focus group consisting of between 6 and 7 participants. Each focus group discussion took place in-person in private conference rooms located at each of the HUB offices. The interviews lasted between 90 and 120 min.

### Data collection

The focus group interviews were conducted in July 2020. Only connectors and interviewers were present at the interviews. At the outset, participants were told that the purpose of the interviews was to gain a better understanding of the community connector program and their work as well as to collect connectors’ perspectives. The interviews were led by two interviewers: the head researcher (LN), is a nursing associate professor who has been involved in the design, development and implementation of the community connector training curriculum; the second interviewer (FC), is a nursing associate professor who has an extensive qualitative research background and substantial experience conducting interviews. Notably, the second interviewer had no prior experience with the community connector program. The semi-structured interview guide consisted of open-ended questions focused on connectors’ field experiences; asset mapping related challenges; and their recommendations on how their work could be better supported by the Ministry of Health and Welfare (see Additional File 1). The interview guide was developed by the head researcher and reviewed by the research team. All interviews were recorded and transcribed verbatim. No further focus groups were conducted following the fourth focus group discussion as we reached data saturation. All identifying information was removed during the transcription process. Demographic characteristics on sex, level of education and age were also collected.

### Data analysis

The focus group recordings were listened to repeatedly and the transcripts were read several times by three researchers (LN, FC, XX). Qualitative content analysis was applied. At first, codes were determined separately by the three researchers. To further ensure credibility, transparency and trustworthiness, the research team reviewed the codes, and proposed themes and subthemes together. Continuous discussion throughout the analysis process was vital to reaching consensus. All data has been translated from Mandarin Chinese into English.

### Ethical considerations

Participants were informed of the purpose of the interview and assured that their anonymity would be protected. All participants were compensated for their time. This study was approved by the Ethics Committee of the Institutional Review Board (IRB) of Chang Gung Medical Foundation, Taoyuan, Taiwan (Reference no. 202001185B0). The IRB did not require participants give written informed consent and granted a waiver as the study involved minimal to no risk to the subjects.

## Results

The participants were predominantly female (88.5%) and participants’ ages ranged between 21 to 53 years old. The majority of participants’ highest earned degree was a bachelor’s degree (53.9%), followed by those with a master’s degree (26.9%) and an associate degree (19.2%). Full details are available in Table 1.

**Table 1:** Characteristics of study participants

Characteristic	Mean (SD); range or n (%)
Sex	
Female	23 (88.5%)
Male	3 (11.5%)
Age (y)	34.9 (9.3); 21–53
Education	
Associate degree	5 (19.2%)
Bachelor’s degree	14 (53.9%)
Master’s degree	7 (26.9%)

Qualitative content analysis revealed three themes, each consisting of subthemes (refer to Table 2). The theme developing community ties consisted of the subthemes: proactive participation and local connections. The second theme cross-organization interactions consisted of the subthemes: resource hoarding and tactful collaboration. The final theme of professional conflicts included the subthemes of task confusion, limited professional stability and poor external recognition.

**Table 2:** Themes and subthemes

Themes	Subthemes
Developing community ties	Proactive participation
	Local connections
Cross-organizational interactions	Resource hoarding
	Tactful collaboration
Professional conflicts	Task confusion
	Limited professional stability
	Poor external recognition

### Developing community ties

#### Proactive participation

Connectors highlighted the importance of proactive participation at community events as a way of cultivating relationships and gaining visibility. By being present at local events, particularly gatherings that exclusively attracted older adult communities, connectors were able to initiate relationships and gain trust.

“…always being around, being visible at these events, it lets them get to know who I am, then once you start going to these activities you start to meet elders, maybe thosewho need support.” (Site B, Participant 1)

Some connectors sought out opportunities, outside of work hours, that allowed them to develop stronger partnerships with community initiatives. By seeking out these opportunities, connectors were able to gain a stronger understanding of local support systems and organizations.

*“Like last time they (community kitchen) added me on their Facebook group, and at lunch that day I saw that they were moving things, so right after they posted that I asked if they wanted help...that was the first time, but in total I’ve helped out three times. Once was on Saturday. I went over to help them distribute lunch boxes...it’s a project that’s gotten backing from the county, so now they are trying to establish it within the city. So I’ve gotten to understand this project more...I think this is really special, so whenever I do have time I go and help out...”* (Site A, Participant 3*)*

#### Local connections

In addition to taking a proactive approach to networking, connectors also drew from their own personal network to cultivate stronger local connections. One connector noted that by drawing on his friend group he was able to gain insight into a local social support network more efficiently.

“It’s because I am local, so it’s pretty much close friends, old drinking buddies…” (Site D, Participant 7)

Connectors also highlighted the importance of developing relationships with other key community members, particularly those who had strong ties with older adult populations. Building these relationships was considered a valuable step in increasing their overall outreach.

*“Every week they have a class where they (elderly community members) will come, so this teacher sees them every week. So in the event that one of them doesn’t come, the teacher will tell us...the teacher has a sense of each elder, and he (the teacher) will see when elders are getting a little worse, and he will make sure to introduce them to us…”* (Site A, Participant 5)

Having personal knowledge and pre-existing community ties gave many connectors a notable advantage. Being able to draw from local contacts was especially important in communities with significant indigenous populations.

*“I think the main thing is that everyone is part of a tribe. There are some elders who have known me from when I was a kid and have watched me grow up...and through this opportunity, I have had the chance to reconnect with these elders...I will work with these elders to understand what they need and what the different tribes could benefit from.”* (Site B, Participant 4)

Understanding how to draw from and cultivate personal local connections allowed connectors to build effective networks, which helped them build trust with older adult populations and gain valuable insight.

### Cross-organizational interactions

#### Resource hoarding

Within the focus group discussions, emphasis was placed on tension between connectors and community organizations, particularly surrounding resource sharing. Connectors described a culture of competition that shaped some of their interactions with local organizations and community leaders. Namely, connectors highlighted that many members of local organizations expressed concerns that connectors would encroach on their work and contributions to the community.

*“They might be worried that their work is going to be eaten up by us”* (Site A, Participant 2)

*“Basically, I don't want them to think that because today that I am, as a HUB member, coming in and going to override them.”* (Site D, Participant 5)

Moreover, these conditions made it difficult for connectors to communicate with local organizations. This made it challenging to fully grasp the quantity and quality of the resources available.

“In actuality, some organizations do have these resources. However, they might feel they want these resources to be more exclusive...and they just don't want to share. Some people will have such thoughts” (Site B, Participant 5)

#### Tactful Collaboration

Connectors described the importance and process of building strong relationships with relevant organizations. This was particularly relevant when working alongside organizations that were unsure and skeptical of the role and impact of connectors. For some connectors this meant being strategic about how much information and work was shared with new collaborators. Namely, it was critical to reassure organizations that their collaboration would not become an additional source of burden.

“If you make them feel that you've just unloaded a lot of work on to them, then you've destroyed the relationship you've built.” (Site C, Participant 5)

Other connectors highlighted the importance of stressing the value of collaboration.

*“So I will be really frank with them, like you don't need to worry, you've been doing it for so many years, people have problems, they will keep looking for you, so you don't need to worry....I can actually help you with this, and it's free. So you can really take advantage of our resources…”* (Site A, Participant 2)

Connectors highlighted the importance of being strategic and subtle when navigating these interactions, particularly when actual clients were involved. Namely, if connectors felt an organization was not able to address the needs of specific clients while connectors were able to provide a more relevant referral, it was critical to approach these interactions strategically.

*“... I will be very cautious...I do need to almost intercept this case a little earlier than them, so in order to do this I need to be very skillful, and graceful...”* (Site D, Participant 5)

Tactfulness was reinforced when describing these collaborations and was seen as a critical trait to ensuring that community groups remain motivated to work alongside them.

### Professional conflicts

#### Task confusion

Within the focus group discussions, task confusion was highlighted. Notably, many connectors expressed that their roles include various responsibilities. This was particularly prevalent in connectors’ confusion surrounding how to compile information on different resources and organizations.

*“When we started the resource inventory, it's really hard, cause sometimes its (the services) very random...for instance...we won't know whether to classify it as fall prevention, nutrition of medication or all three...this will result in a lot of redundancy and I don't know if this is actually good or bad”* (Site C, Participant 4)

Other connectors highlighted that asset mapping was challenging, particularly at the outset, when they could not rely on significant community networks.

“At first when we started with the resource inventory, it was really hard to figure outhow to start the process… because you don't really know people..” (Site A, Participant5)

In addition to completing asset mapping, connectors also expressed that their interactions with service users also presented instances wherein their professional responsibilities were not clear. Namely, the line between case management and resource provider was occasionally blurred.

“…Frankly speaking I am a little bit lost… is that I am supposed to link them with resources or is it that I have to help them solve their problems? It becomes that I've come to adopt a social worker role, it feels like it's in that domain…I do want to help them, however I am worried about what can be done. Sometimes I will feel confused about my role, right now it feels like that.” (Site D, Participant 2)

#### Limited professional stability

Beyond the obstacles they experienced in their roles, many community connectors highlighted that part of the challenges they faced were the result of limited professional stability. Many noted that their roles, inherently, embodied a degree of planned obsolescence.

"The directors ideal is, when there isn't the HUB, the organization will know how pass on cases…they won't need to go through us" (Site D, Participant 2)

“Today, if we are going to tell the community chief, if you have a service user with dementia you can probably use these resources…the next time the community chief encounters a similar case he won't have to rely on the HUB…if in the future, the Ministry of Health and Welfare will evaluate the resource platform, and all the resources will be uploaded, then what will the HUB be doing in the future? This is the problem.” (Site C, Participant 2)

For some connectors, the lack of long-term stability made it especially difficult to maintain professional motivation.

“…(L)ast year none of us were here, and by next year there will be new people, and next year it will be tough because the plan will be ending, so why bother.” (Site B, Participant 2)

#### Poor external recognition

Finally, a challenge faced by connectors was the widely held belief that their roles and contributions, as connectors, were not fully understood. This lack of clarity, surrounding the capacities of connectors’ work, created obstacles in the communication and cooperation between connectors and community leaders.

*“In terms of connecting, because the community's leaders will have certain expectations of us. They will think we are direct service providers, but we will deal with it by telling them it like this...let's say we go traveling, there may be a tourist center, we basically have the same kind of role as a tourist center"* (Site B, Participant 1)

This lack of understanding also impacted their interactions with service users. Namely, some service users expressed frustration as they expected that connectors would provide more direct support.

*“...We aren't supposed to help them directly solve problems, but we are supposed to provide resources. When the public hears this, they just think - ‘so all they do is just talk to us on the phone?’...”* (Site C, Participant 3)

## Discussion

Our exploratory qualitative research study contributes new insight to the wider field of public and community health research, particularly as it relates to workers who engage directly with community members as well as those who are tasked with large-scale community projects, which require strong community ties and local knowledge. Our findings add considerable insight into the steps taken to cultivate partnerships that shape community connectors’ work. Notably, the importance of active and consistent participation in community events, drawing on existing community ties and strategic professional relationships were identified as critical. Indeed, the process of how connectors build relationships within their community should not be overlooked. Existing research indicates that being able to cultivate connections, based on community trust, is integral to the success of community health initiatives [20, 21]. Moreover, these strong relationships between community connectors and older adult service users can have a positive impact and affect service users' willingness to access social services [22]. However, across the literature on community connectors, interpersonal dynamics and steps taken to promote community outreach are not often explored. Namely, the ability for community connectors to gain community confidence is often presumed or deemed a prerequisite to community connectors’ involvement [23–25].

Beyond highlighting the steps taken to develop trust and connection, the study also uncovered that, in order to build community partnerships, connectors would work during the weekends and evenings. This finding is important insofar as it illuminates that initiatives that would similarly hire community connectors on a full time basis, would benefit from instituting flexible working schedules as well as ensuring, beforehand, that staff are able to work these hours. When considering the multifaceted nature of community connectors’ work, the need for flexibility is evident. In George et al.’s (2017) analysis of community health workers’ experience in Delhi, the value of flexible schedules and autonomy in their daily work were emphasized in discussion of their roles [26]. Moreover, research on healthcare sector workers has found that flexible work arrangements can have a positive impact on workers’ health [27].

In addition, our findings also illuminate the importance of ensuring that community connector teams represent, as much as possible, the communities they aim to support. For instance, when working with indigenous communities, it was especially critical to have community connectors who are indigenous and have grown up locally. Previous studies have also shown that having a diverse workforce, particularly when working alongside marginalized communities, can help foster stronger community relationships and lead to better utilization of direct health services and public health resources [28, 29]. Future research may benefit from imagining how instituting further diversity—across markers such as age, class and ability—may also serve to better promote community connectors’ work and aims.

The findings from our study also reveal that connectors face the unique challenge of navigating an obscure world of nonprofits and informal community-run organizations. Connectors highlighted that, in several instances, organizations that work with older adult populations expressed overall hesitancy to share resources and to work alongside connector teams. Indeed, efforts to collaborate between nonprofits and local governments can be particularly complex, due to low trust, differing priorities and dissimilar management and monitoring structures [30–32]. Notably, it has been found that, amongst Taiwan nonprofit organizations, concerns surrounding finances and performance have been identified as reasons that deter nonprofits from full transparency [33]. As a tactic to navigate a culture of skepticism and lack of transparency directed towards intersectoral collaboration, the importance of cultivating tactful and strategic relationships has been highlighted by connectors. Hu et al.’s (2020) research has similarly emphasized the role strategic professional partnerships and friendship play in terms of enhancing interorganizational collaboration efforts [34].

Finally, the findings in this study highlight that part of the challenges faced by connectors stem from an ambiguous sense of their professional role and a lack of professional security. In the focus group discussions, connectors highlighted the stress surrounding the multifarious nature of their roles. Their challenges echo the experiences of other community health workers, who similarly juggle various responsibilities and struggle with the ambiguous nature of their titles, coupled with the public’s lack of understanding of their work [35]. Indeed, the impact of poor external understanding alongside lack of professional security should not be taken lightly, as these circumstances can greatly impact work and organizational commitment [36]. Namely, existing research shows that a poor sense of professional identity and professional role can have a negative impact on workplace dynamics and interprofessional collaboration [37]. In existing research on community connectors, challenges surrounding professional identity have not been explored, as many of these initiatives tend to recruit volunteers who do not receive extensive training. As such, it is recommended that community connector initiatives, with full time paid staff, strive to ensure that professional identities and the mandates are clearly articulated, in order to avoid unnecessary confusion and to maintain strong organizational commitment.

## Conclusion

This study explored the experiences of community connectors working across various sites in Taiwan. Our findings add to existing literature on the development of on-the-ground public health outreach initiatives. These findings are especially pertinent to initiatives that seek to employ staff on a full-time basis and those that aim to prioritize resource management over case management. Namely, this study points to the necessity of providing these public health workers with a greater degree of professional support, in order to ensure that they can adapt successfully to their roles and the communities that they work within. That said, this study has several limitations. Firstly, the findings are specific to the context of Taiwan. Nevertheless, similar public health initiatives outside of Taiwan can still draw from our findings. Secondly, this study had a small sample size (26 participants from four sites); more participants would have contributed further insight and made our findings more generalizable. That said, our study included connectors from diverse sites across Taiwan, which does support the generalization of our results. Community connector and related public health worker initiatives can draw from our findings to examine and anticipate challenges faced by on-the-ground public health workers.

## Supplementary Information

**Additional file 1.** Interview guide.

## Data Availability

The datasets generated during and analyzed during the current study are available from the corresponding author on reasonable request.

## Abbreviations

IRB: Institutional Review Board
SD: Standard deviation
Age (y): Age (years)
