# Supplementary material for: Challenges faced by community connectors: lessons learned from a Taiwan public health initiative
Source: BMC Geriatr. 2022 Nov 19;22:876. doi: 10.1186/s12877-022-03565-8 (PMC9675122; doi:10.1186/s12877-022-03565-8)
Supplement: Supplementary file 1 — Additional file 1. Interview guide. [file 12877_2022_3565_MOESM1_ESM.docx]

INTERVIEW GUIDE

Part 1: Developing an inventory of community resources: personal experiences and areas that need further attention

1. What has been your experience, both good and bad, with the resource inventory process (i.e., asset mapping)?
2. What are the main reasons for some of the challenges you have faced during the resource inventory process? How did you manage these challenges?
3. What kind of assistance do you think the National Health Administration can provide? What are your suggestions?

Part 2: Connecting community resources: personal experiences and areas that need further attention

1. What has been your experience, both good and bad, connecting community members with resources?
2. What do you think are the main reasons that you have encountered difficulties connecting users to community resources? How did you manage these challenges?
3. What kind of assistance do you think the National Health Administration can provide? What are your suggestions?
